# Influence of the duration of type 2 diabetes mellitus on colorectal cancer outcomes

**DOI:** 10.1038/s41598-023-40216-3

**Published:** 2023-08-10

**Authors:** Magdolna Herold, Attila Marcell Szasz, Gyongyver Szentmartoni, Emoke Martinek, Viktor Madar-Dank, Andras Jozsef Barna, Reka Mohacsi, Aniko Somogyi, Magdolna Dank, Zoltan Herold

**Affiliations:** 1 Department of Internal Medicine and Hematology, Semmelweis University, Budapest, 1088 Hungary; 2 Division of Oncology, Department of Internal Medicine and Oncology, Semmelweis University, Budapest, 1083 Hungary; 3 Department of the Institute for Dispute Resolution, New Jersey City University, Jersey City, NJ 07311 USA; 4 Department of Obstetrics and Gynecology, Saint Pantaleon Hospital, Dunaujvaros, 2400 Hungary

**Keywords:** Endocrinology, Oncology

## Abstract

Type 2 diabetes mellitus (T2DM) is a progressive disease, which affects colorectal cancer (CRC) survival. However, data on the relationship between CRC survival and T2DM duration is scarce and controversial. A retrospective observational study was conducted. Sub-cohorts were created based on the duration of T2DM as follows, ≤ or > 5/10/15/20 years. 204 of the 817 (24.95%) included study participants had T2DM at any point of CRC. 160 of the 204 CRC + T2DM patients had detailed T2DM duration data. At the time of CRC diagnosis, 85, 50, 31, and 11 patients had T2DM for > 5/10/15/20 years, respectively, which increased to 110, 71, 45, and 17 during the course of the study. Despite constant glycated hemoglobin values throughout the study, shorter overall and disease-specific survival times were observed for the > 5/10/15 years cohorts and longitudinal survival modeling techniques confirmed the significant effect of T2DM duration in all cohorts. While in the first 3 years after CRC diagnosis, the best survival was found for the ≤ 5 years cohort, all diabetes cohorts had the same survival thereafter. T2DM duration affected CRC survival significantly, therefore, a closer follow-up of this sub-populations is suggested.

## Introduction

Colorectal cancer (CRC) is the third most common cancer type^1^, which has several known risk factors, including type 2 diabetes mellitus (T2DM). Diabetes mellitus is one of the most prevalent diseases of our time with an estimated 573 million diabetes patients around the world, of which ~ 90% have T2DM^2^. CRC and T2DM share several risk factors, including but not limited to, obesity and sedentary lifestyle, increased inflammatory cytokines, insulin resistance, hyperinsulinemia, and various genetic factors^3,4^. Moreover, CRC is known to have an approximately 50% higher incidence in those with T2DM^5^, and higher all-cause mortality and cancer-specific mortality were also observed in those patients suffering from both diseases, compared to CRC patients without T2DM^6^.

Although T2DM is characterized as a progressive disease, e.g., the number of diabetes-related complications and co-morbidities increases with its duration^4,7^, previous studies investigating the relationship between CRC and T2DM duration found controversial results. Two recent studies^8,9^ have reported that cancer-specific mortality is not affected by the duration of diabetes in CRC^8,9^, neither in breast and prostate cancer nor if all sites were analyzed together^8^. However, all-cause mortality has been affected by diabetes duration in both studies^8,9^. The opposite has been also reported, e.g., cancer-specific mortality has been significantly affected if T2DM is present for ≥ 10 years^10^, while another^11^ study could justify the same for only female T2DM + CRC patients. It has to be noted, however, that basically all of these studies performed single-time survival analyses only, and to our knowledge, no longitudinal analysis has been performed investigating this aspect previously. Therefore, a longitudinal retrospective study was conducted to determine whether longer T2DM durations are associated with shorter CRC survival and whether this risk increases with the course of CRC and/or T2DM. Moreover, secondary questions of the study were how the various routine laboratory parameters, such as complete blood count or liver enzymes change over time in CRC patients with or without T2DM.

## Results

The retrospective data of 817 CRC patients were collected. 204 of the 817 study participants (24.97%) had T2DM. Of these 204 patients, 160 had (78.43%) details about the duration of T2DM: 122, 24, and 14 patients were diagnosed with T2DM prior to, at the time of, and after the diagnosis of the tumor, respectively. CRC patients with T2DM were 4 years older on average (*P* < 0.001), compared to those without T2DM. 4 (2%) and 67 (10.9%) patients were under the age of 50 years within the CRC with and without T2DM groups, respectively. T2DM + CRC was more common in men (*P* < 0.001), and—as expected—hypertension and major cardiovascular (mCV) events occurred more often in the T2DM groups. CRC patients with T2DM had higher BMI (non-T2DM: 26.31 ± 4.84 kg/m^2^; T2DM: 28.37 ± 4.59 kg/m^2^; *P* < 0.001). Moreover, right-sided tumors were more common in the T2DM + CRC women (46.9% vs. 33.9%; *P* = 0.001), than in those without T2DM, where the sidedness-to-sex ratio did not differ. Medical history and clinicopathological data of study participants with or without T2DM are summarized in Table 1.

**Table 1:** Medical history and histopathological data of study participants.

Clinicopathological characteristics	Without T2DM (*n* = 613)	With T2DM (*n* = 204)	*P* value
Age (year)	64.42 ± 11.61	67.85 ± 8.27	< 0.001
Male:female ratio	317:296 (51.7%:48.3%)	139:65 (68.1%:31.9%)	< 0.001
Irresectable tumor	50 (8.2%)	17 (8.3%)	1.000
AJCC staging^45^ at tumor diagnosis:			0.882
Stage I	67 (10.9%)	(17.2%)	
Stage II	159 (25.9%)	24.5%)	
Stage III	134 (21.9%)	48 (23.5%)	
Stage IV	253 (41.3%)	71 (34.8%)	
Regional lymph node metastasis	279 (45.5%)	92 (45.1%)	1.000
Distant metastases:			1.000
Synchronous	253 (41.3%)	71 (34.8%)	
Metachronous	75 (12.2%)	22 (10.8%)	
Location of the tumor^46^:			1.000
Left-sided	416 (67.9%)	0.1%)	
Right-sided	183 (29.9%)	56 (27.5%)	
Both (multiplex tumor)	14 (2.3%)	5 (2.5%)	
Neoadjuvant chemotherapy	68 (11.1%)	18 (8.8%)	1.000
Final lineage of chemotherapy			1.000
Adjuvant only/First line	281 (45.8%)	42.6%)	
Second line	71 (11.6%)	25 (12.3%)	
Third line or above	81 (13.2%)	21 (10.3%)	
Radiotherapy			1.000
Preoperative	46 (7.5%)	7.8%)	
Postoperative	49 (8.0%)	14 (6.8%)	
Pre- and postoperative	4 (0.7%)	1 (0.5%)	
Use of biological agents^a^	182 (29.7%)	42 (20.6%)	0.147
Use of regorafenib or trifluridine/tipiracil	43 (7.0%)	10 (4.9%)	1.000
Medical history			
Hypertension	370 (60.4%)	184 (90.2%)	< 0.001
Major cardiovascular event(s)^b^ prior to CRC	88 (14.4%)	63 (30.9%)	< 0.001
Thyroid disease (in euthyroid state)	57 (9.3%)	25 (12.3%)	1.000
Appendectomy	93 (15.2%)	47 (23.0%)	0.160
Cholecystectomy	82 (13.4%)	39 (19.1%)	0.581
Medications			
Antihypertensive therapy	345 (56.3%)	174 (85.3%)	< 0.001
Statin therapy	86 (14.0%)	63 (30.9%)	< 0.001
Aggregation inhibition	85 (13.9%)	68 (33.3%)	< 0.001

Continuous and count data are presented as mean ± standard deviation and number of observations (percentage), respectively.

^a^Bevacizumab, cetuximab, and panitumumab. ^b^Myocardial infarction, stroke, transient ischemic attack, pulmonary embolism, coronary artery bypass grafting, and/or stent implantation. *AJCC* American Joint Committee on Cancer, *T2DM* type 2 diabetes mellitus.

Patients were also grouped based on whether they have had T2DM for over 5/10/15/20 years or less: (1) ≤ 5 years (*n* = 75) versus > 5 years (*n* = 85); (2) ≤ 10 years (*n* = 110) versus  > 10 years (*n* = 50); (3) ≤ 15 years (*n* = 129) versus  > 15 years (*n* = 31); and (4) ≤ 20 years (*n* = 149) versus > 20 years (*n* = 11). Incidental T2DM and patients with only prediabetes at the time of tumor diagnosis were assigned to the ≤ 5/10/15/20 years groups. The clinicopathological parameters of the patients were compared, and except for some differences in age and the number of previous mCV events, no further differences could be justified. In general, patients in the ≤ 5/10/15 years groups were 4–5 years younger (≤ 5 vs. > 5: 64.7 ± 8.2 years vs. 69.9 ± 7.8 years, *P* < 0.001; ≤ 10 vs. > 10: 66.2 ± 8.1 years vs. 70.4 ± 8.3 years, *P* = 0.004; ≤ 15 vs. > 15: 66.7 ± 8.5 years vs. 70.6 ± 7.2 years, *P* = 0.011), except for the ≤ 20 versus  > 20 groups (*P* = 0.846). Previous mCV events occurred less often in the ≤ 5 (22.7% vs. 35.3%, *P* = 0.086), ≤ 10 (23.6% vs. 42.0%, *P* = 0.024), and in the ≤ 15 (24.8% vs. 48.4%, *P* = 0.015) groups, while no difference was found in the ≤ 20 versus > 20 comparison (*P* = 0.302).

### Comparison of laboratory parameter changes in patients with and without T2DM

**Figure 1:**
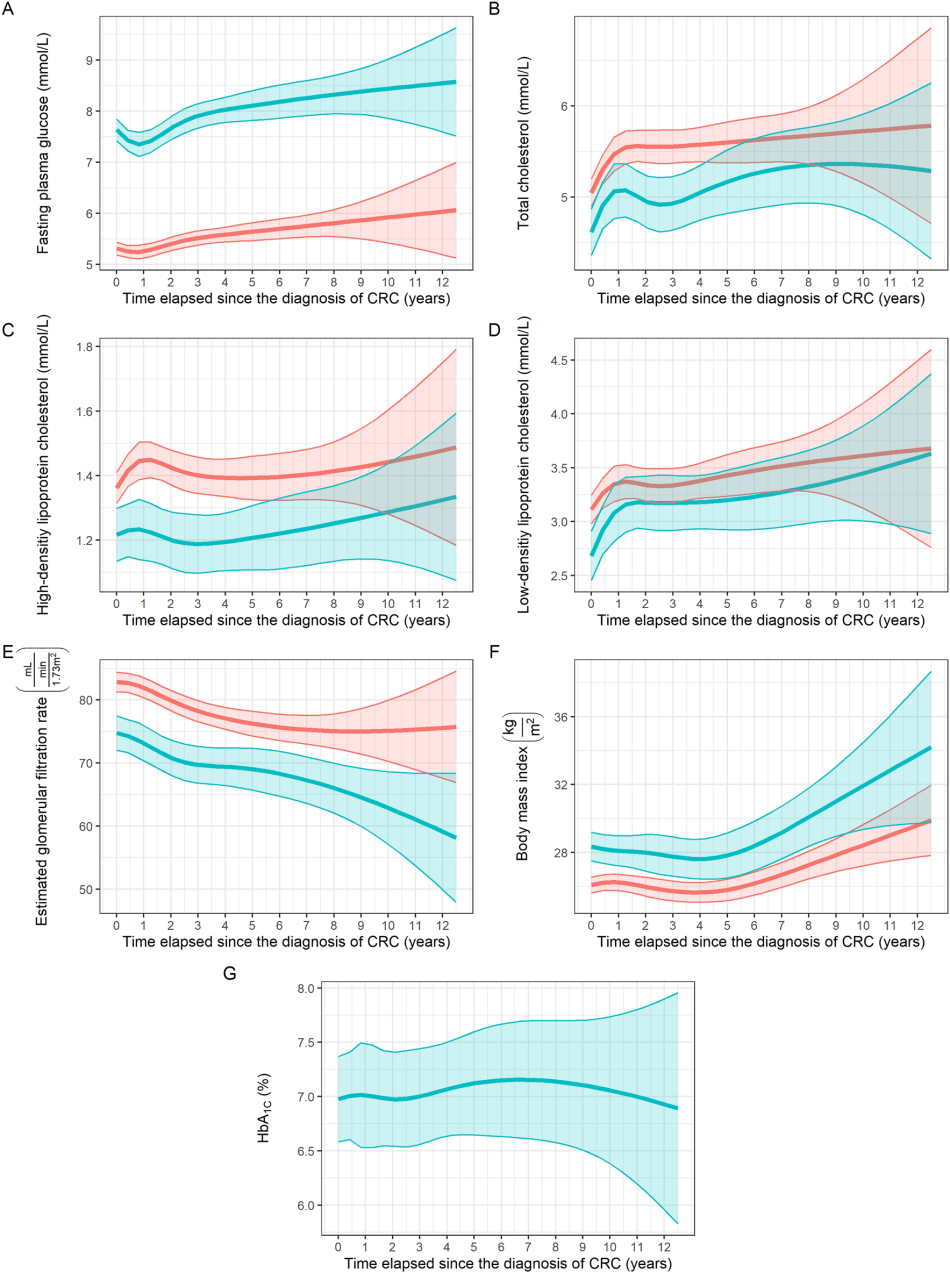
Changes in the average fasting plasma glucose (**A**), total– (**B**), high-density lipoprotein– (**C**), low-density lipoprotein cholesterol (**D**), estimated glomerular filtration rate (**E**), body mass index (**F**) and glycosylated hemoglobin (HbA_1C_; **G**) values of the two study cohorts during the observation period. Significant constant differences between the two cohorts could be justified in all parameters (**A**–**F**). Optimal fasting glucose, HBA_1C_, total–, high-density lipoprotein–, and low-density lipoprotein cholesterol lipid values for T2DM patients are between 4.0 and 6.5 mmol/L, < 7.0%, < 4.0 mmol/L, > 1.0 mmol/L, < 2.0 mmol/L, respectively. Green and red colors represent colorectal cancer patients with and without type 2 diabetes mellitus, respectively. Thick lines and ribbons represent the predicted values and their 95% confidence intervals at the specific timepoints, respectively.

Longitudinal analysis of changes in the various routine laboratory parameters was first performed in those CRC patients with or without T2DM to identify any differences between the two cohorts. Due to the known effects of prediabetes on metabolic parameters^4,12,13^, those patients who developed T2DM around (6 months prior to diagnosis) or after the diagnosis of CRC, were also included in the T2DM cohort throughout the whole observation. The following differences and/or trends could be identified: Glycosylated hemoglobin (HbA_1C_) of T2DM patients was constant throughout the whole study (*P* = 0.935, Fig. 1G). Fasting plasma glucose (*P* < 0.001; Fig. 1A) and serum creatinine (*P* < 0.001) were constantly higher, while total cholesterol (*P* = 0.004; Fig. 1B), high-density lipoprotein cholesterol (HDL; *P* = 0.003; Fig. 1C) and low-density lipoprotein cholesterol (LDL; *P* = 0.002; Fig. 1D), and estimated glomerular filtration rate (eGFR; *P* < 0.001; Fig. 1E) were constantly lower in the T2DM cohort throughout the whole study, compared to the non-T2DM cohort. No further statistical differences in the other laboratory parameters could be justified. It also has to be mentioned that the body mass index of the CRC + T2DM patients was constantly significantly higher throughout the whole observation period (*P* < 0.001; Fig. 1F).

### Comparison of laboratory parameter changes in patients with different T2DM durations

It was also evaluated whether the duration of T2DM is associated with any longitudinal changes. At the end of our observation, 110, 71, 45, and 17 patients had a T2DM duration greater than 5, 10, 15, and 20 years, which was a 25, 21, 14, and 6 head increase compared to the occurrences seen at the time of CRC diagnosis, respectively. Therefore, during modelling, we did not strictly set any groups for the patients from start to finish, but changing groups was allowed for every patient during the course of the study, according to the actual duration of T2DM. This analysis was performed only in the subset of patients, who had any data on the duration of T2DM (*n* = 160), and the following subgroups were created: ‘≤ 5 years’, ‘5–10 years’, ‘10–15 years’, ‘15–20 years’ and ‘> 20 years’. This approach was used in order to be able to compare the various sub-cohorts directly.

**Figure 2:**
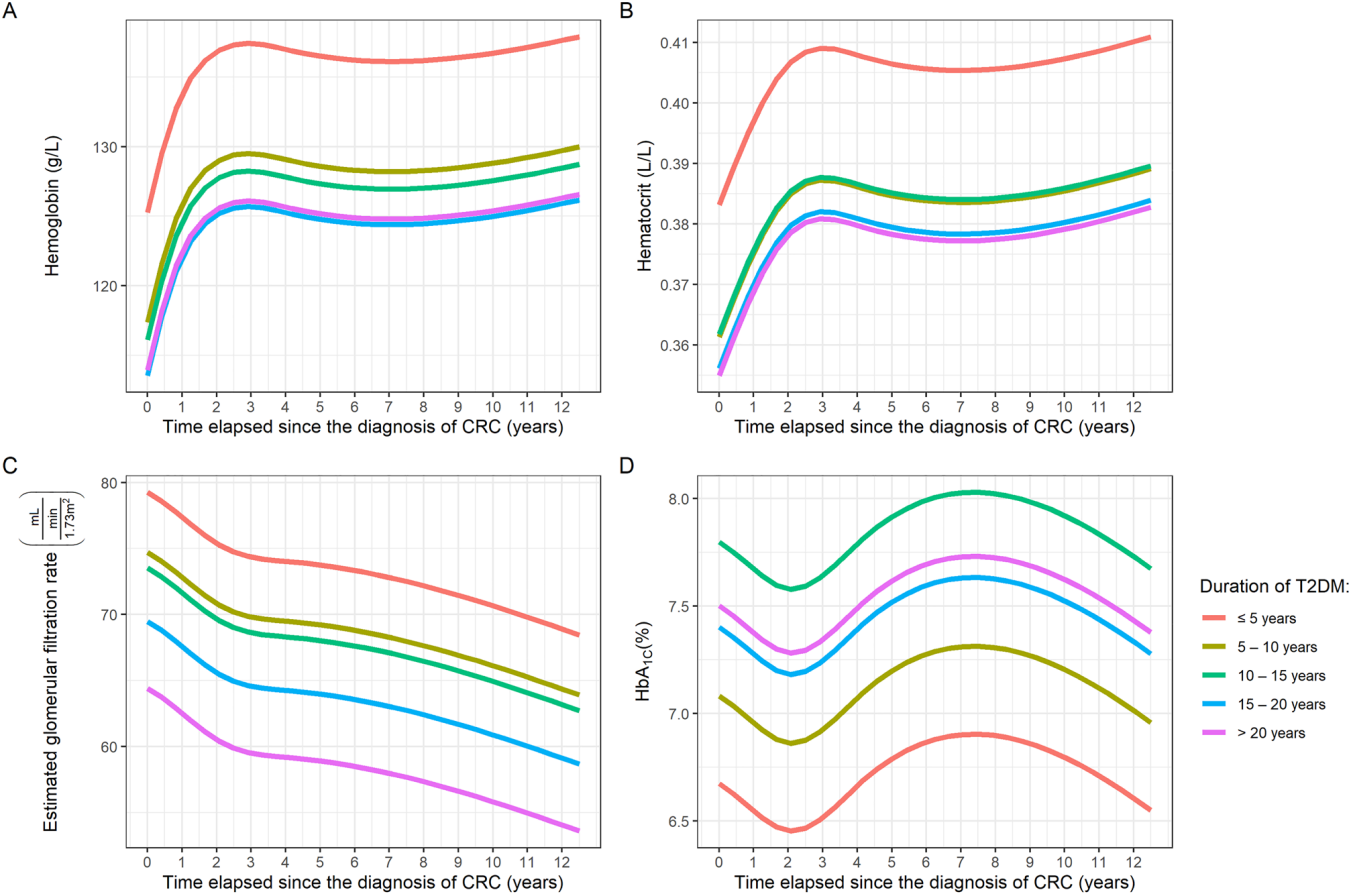
Changes in the average hemoglobin (**A**), hematocrit (**B**), estimated glomerular filtration rate (**C**) and glycosylated hemoglobin (HbA_1C_; **D**) values of the study cohorts with different duration of type 2 diabetes mellitus (T2DM), during the whole observation period.

Constantly lower hemoglobin was found in those patients having T2DM between ‘5–10 years’ (*P* = 0.035), ‘10–15 years’ (*P* < 0.001), and ‘15–20 years’ (*P* < 0.001), compared to those with a T2DM duration ≤ 5 years. Moreover, a significant difference between the ‘5–10 years’ and ‘15–20 years’ groups was also found (*P* = 0.030; Fig. 2A). Similar trends were found in the hematocrit values of patients. Hematocrit was significantly lower throughout the study in the ‘5–10 years’ (*P* = 0.026), ‘10–15 years’ (*P* = 0.003), and ‘15–20 years’ (*P* < 0.001) cohorts, compared to those in the ‘≤ 5 years’ cohort (Fig. 2B). eGFR of the ‘5–10 years’ (*P* = 0.027) and the ‘> 20 years’ (*P* = 0.043) cohorts was significantly lower than that of the ‘≤ 5 years’ cohort (Fig. 2C). Tendentiously higher HbA_1C_ levels were found in those patients with longer T2DM durations (*P* = 0.061; Fig. 2D).

### Survival results

#### Conventional survival modeling results

**Figure 3:**
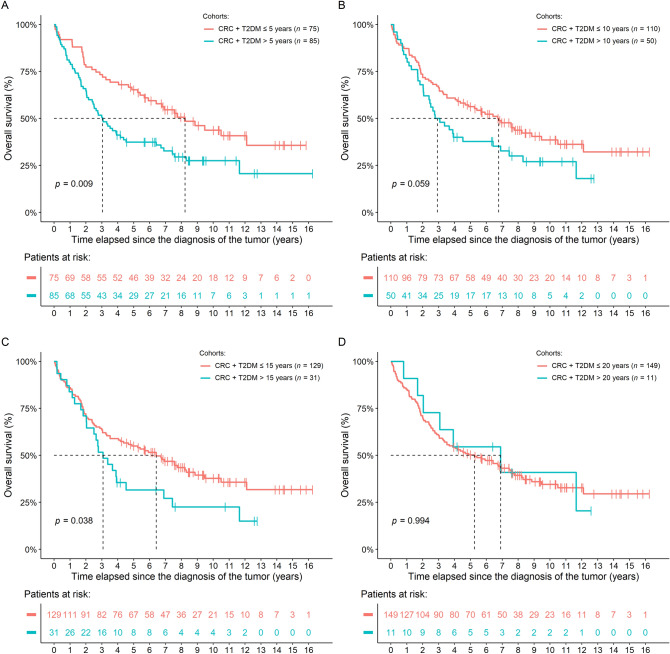
Differences in the overall survival of patients with a type 2 diabetes mellitus duration of (**A**) ≤ 5 years versus  > 5 years, (**B**) ≤ 10 years versus  > 10 years, (**C**) ≤ 15 years versus  > 15 years, and (**D**) ≤ 20 years versus  > 20 years. While in the first 3 comparisons patient survival was significantly or marginally worse, in the last comparison no difference could be justified.

Survival data of patients were first investigated using conventional survival modeling techniques. All models were adjusted for the year when the tumor was diagnosed. No univariate effect of T2DM alone could be justified [overall survival (OS): *P* = 0.421; disease-specific survival (DSS): *P* = 0.483]. If the T2DM cohort was analyzed separately, it was found that in those patients with a T2DM duration > 5 years both OS (hazard rate (HR): 1.794, 95% confidence interval (CI): 1.159–2.778, *P* = 0.009) and DSS (HR: 1.718, 95% CI: 1.053–2.804, *P* = 0.030) were significantly shorter. A similar tendency was found when the CRC + T2DM patients were grouped into ‘≤ 10 years versus > 10 years’ (OS: *P* = 0.059; DSS: *P* = 0.029) and ‘≤ 15 years versus > 15 years’ (OS: *P* = 0.038; DSS: *P* = 0.010) cohorts. However, no difference between the ‘≤ 20 years versus > 20 years’ cohorts could be justified neither in OS (*P* = 0.994), nor in DSS (*P* = 0.554; Figs. 3 and S1, Table S1).

In addition to the above, it was evaluated how these cohorts compared to non-T2DM CRC patients. It was found that, except for the patients with or without T2DM > 20 years, the ≤ 5/10/15/20 years groups had basically the same survival tendencies as the non-T2DM CRC patients, while the “greater than” groups had significantly worse OS (non-T2DM vs. T2DM > 5 years: *P* = 0.009; non-T2DM vs. T2DM > 10 years: *P* = 0.051; non-T2DM vs. T2DM > 15 years: *P* = 0.024), and mostly tendentiously worse DSS (Figs. S2 and S3, Table S2).

Furthermore, using a multivariate survival model we also investigated whether there is a connection between the age of patients and the exact duration of T2DM (in years) regarding the survival of patients. It was found that age had no effect on patient survival (*P* = 0.330), but the longer T2DM durations predicted significantly shorter survival times (HR: 1.036; 95% CI: 1.006–1.066; *P* = 0.017). Interpretation of the latter: for each year lived as a T2DM patient, the risk for shorter CRC survival is 1.036 raised to the power of duration of T2DM in years (1.036^T2DM duration^).

A second set of multivariate models were also performed, where in addition to the T2DM subgroups with or without the non-T2DM subjects, age, hypertension, mCV event(s) prior to CRC, thyroid diseases, appendectomy, cholecystectomy, and staging were included as additional explanatory parameters. Staging had the strongest influence on survival in all models, it was the only significant effector with *P* < 0.001. Therefore, for further testing the effects of these other parameters, staging was removed from the following survival models. After the removal of staging from the explanatory parameters of the models, the same tendencies were obtained as detailed previously: T2DM patients with shorter disease duration had better survival. None of the other parameters had significant effect over survival if only the T2DM patients were included in the models (Table S3). However, if all study participants were included, all patients with hypertension had an increased risk for a shorter survival (Table S4).

#### Longitudinal survival modeling results

**Figure 4:**
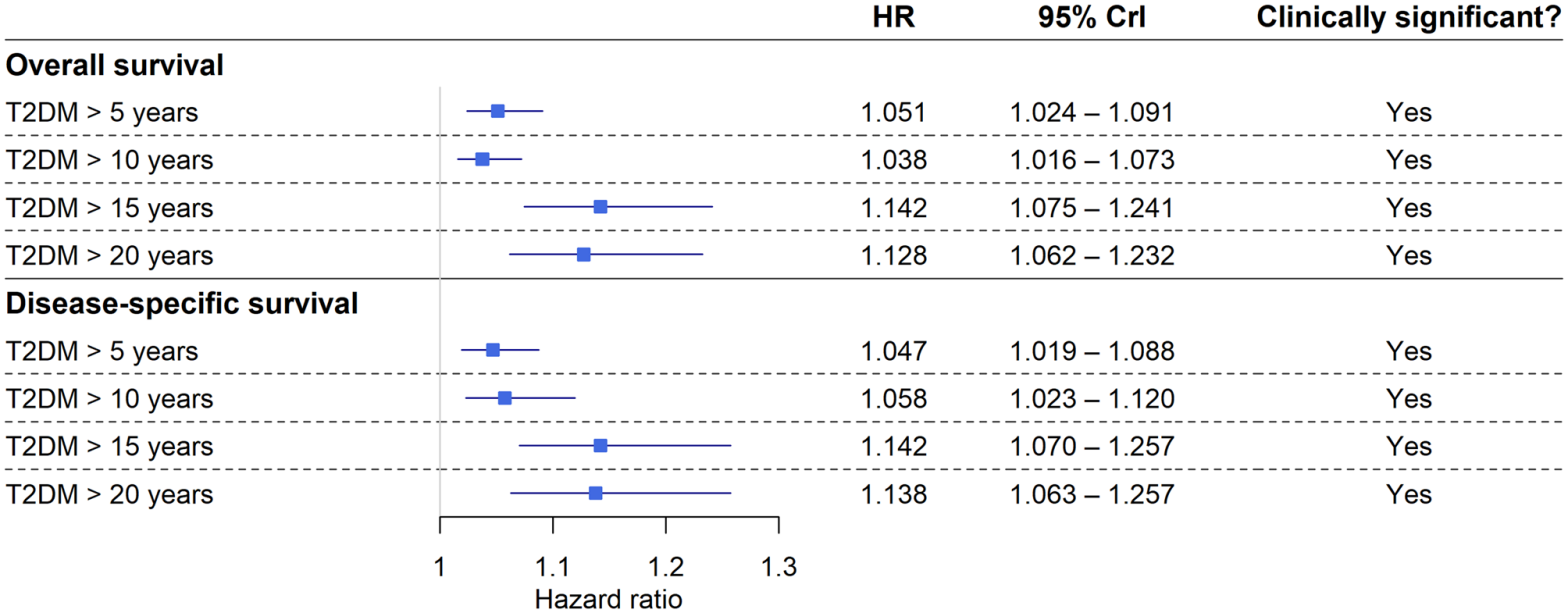
Results of the Bayesian joint survival models. The effect of the T2DM duration greater than 5/10/15/20 years was investigated whether the increase of their occurrence over time is associated with shorter colorectal cancer survival, and a clinically significant association was found in all four cases. *CrI* credible interval, *HR* hazard rate. *Note*: The results computed for disease-specific survival are somewhat distorted due to the lack of methods for competing risk calculation via joint survival modeling. Therefore, non-CRC related deaths were marked as censored events, which might have resulted in some overestimation of the HRs and their 95% CrIs.

**Figure 5:**
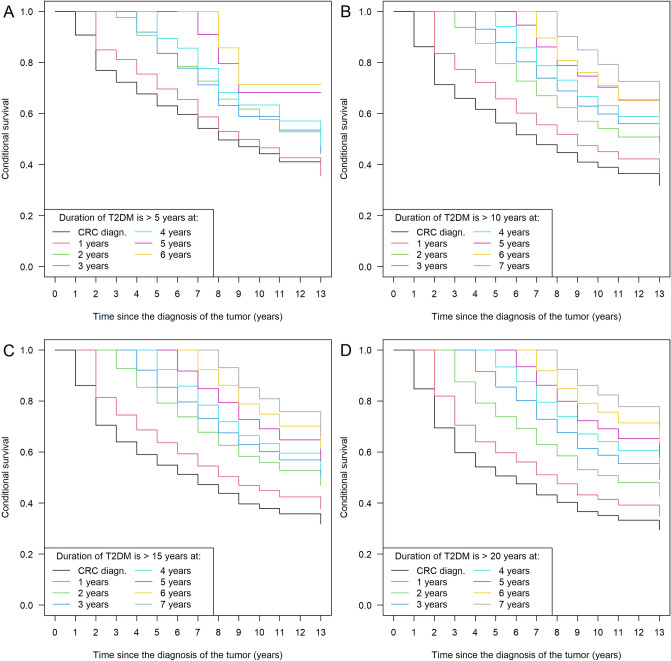
Conditional overall survival of colorectal cancer (CRC) patients, who synchronously had type 2 diabetes mellitus (T2DM) for (**A**) > 5 years, (**B**) > 10 years, (**C**) > 15 years, and (**D**) > 20 years. Using this method, it could be calculated whether the later the 5/10/15/20 years duration of T2DM occurs, the better the survival of the patients is. For example, interpretation of (**A**): those CRC patients who wad T2DM > 5 years at the time of CRC diagnosis (black line) are expected to have the worst survival, followed by those patients whose T2DM duration exceeds 5 years 1-year post-CRC diagnosis (red line). All of the remaining survival curves represent those conditions when the duration of T2DM reaches 5 years 2-, 3-, 4-, 5- and 6-years post-CRC diagnosis. *Note*: CRC patients without T2DM could not be included in this type of analysis.

**Figure 6:**
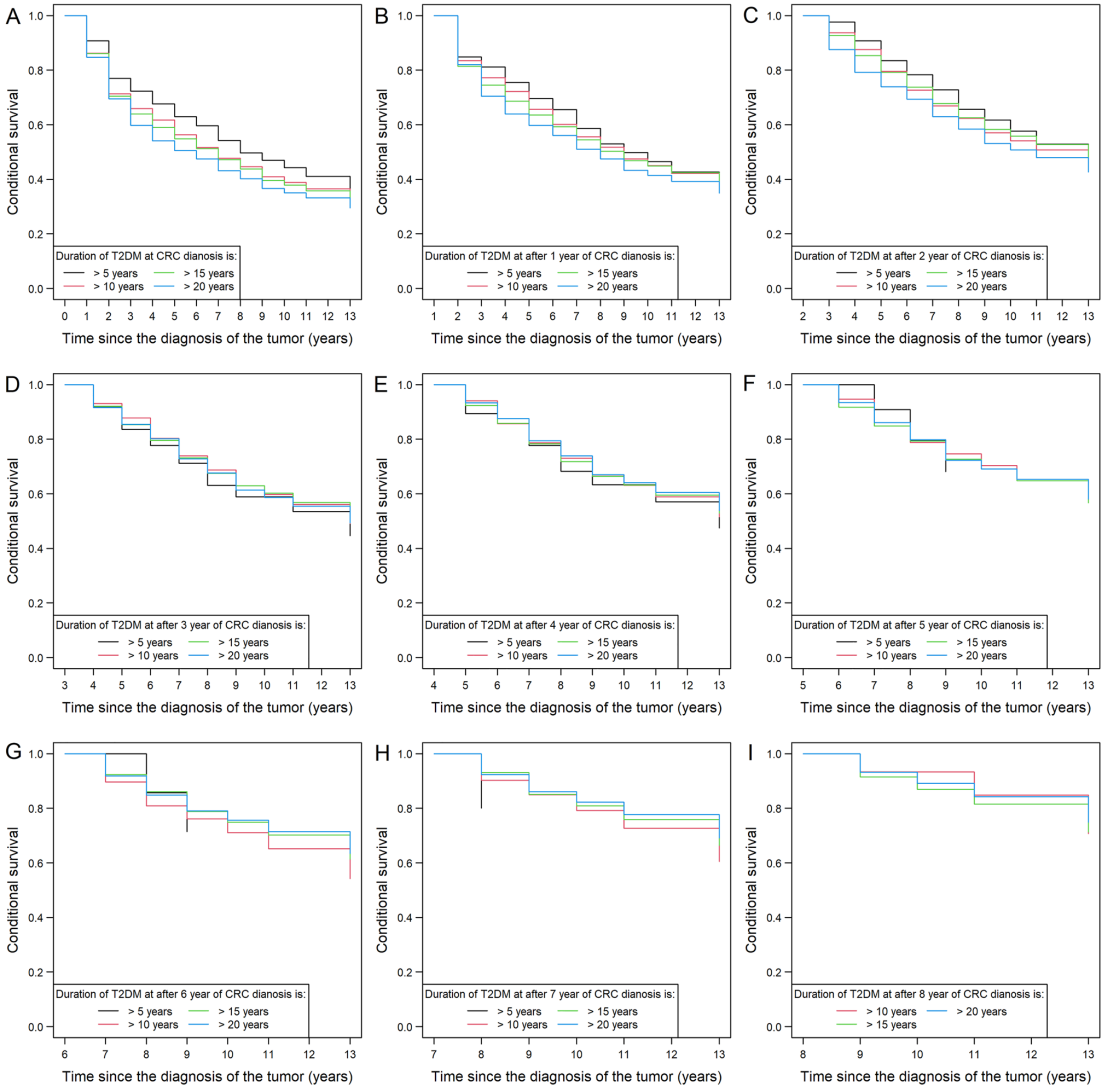
Conditional overall survival of colorectal cancer (CRC) patients, who synchronously had type 2 diabetes mellitus (T2DM) for 5/10/15/20 years. By comparing the conditional survival curves of the 4 sub-cohorts it was found that around the 3rd year after the diagnosis of CRC, the positive effect of the shorter T2DM durations over patient survival became less prominent, and the four sub-cohorts are basically the same thereafter. *Note*: CRC patients without T2DM could not be included in this type of analysis.

The survival data of patients were further analyzed using joint survival modeling techniques. An increasing risk for shorter survival times was found with the increase in the occurrence of longer T2DM durations (Fig. 4), and this trend could be observed in every T2DM sub-cohorts. Moreover, using conditional survival we could further strengthen the observations of the joint models; the later the 5-year diabetes occurred after the CRC diagnosis, the better the patients’ survival was (Fig. 5). It has to be noted, however, that if the conditional survival curves were visually compared, the advantageous effect of shorter T2DM durations was no longer so obvious after the 3rd year of CRC diagnosis (Fig. 6).

Due to the lack of joint and/or conditional survival models for competing risks, we were only able to accurately examine OS. To examine DSS, we could only use a method that gives slightly distorted results: those patients who died of other causes other than CRC were censored. Using this technique, the same results could be obtained, as detailed above (Figs. 4, S4, and S5). The only difference was that in the case of DSS, the equalization of patient survivals of the different T2DM durations occurred somewhat earlier (Fig. S5), in comparison to OS.

## Discussion

Although most up-to-date researches are in agreement that T2DM is a negative effector of CRC survival^6^, there are also publications that have suggested that T2DM might affect overall survival only, and the connection between the two diseases is somewhat less direct^14,15^. In the last decades, several mechanisms were found that may link the two diseases together, including but not limited to obesity and sedentary lifestyle^16,17^, hyperinsulinemia, hyperglycemia, and increased levels and/or genetic variants of insulin-like growth factor-1^18–20^. Moreover, women and men with T2DM have higher tendencies to develop right-sided and left-sided CRC, respectively, compared to the non-T2DM population^21^. This observation could be also confirmed in the current study. Lately, several studies have suggested that CRC patients with T2DM may receive less often CRC-specific treatments^15,22–25^. In the current study, we could not observe the same, the proportions of patients receiving various kind of oncological treatments were the same in the two tumor cohorts.

T2DM is characterized as a progressive disease^4,7^, therefore, one could assume that those T2DM patients with a longer T2DM duration will also have worse CRC survival. However, the situation is not so black and white, for example, adherence to therapy is one if not the most important factor affecting T2DM progression^26^. E.g., patients with better and worse adherence have (usually) less and more progressed disease, respectively, even if they have T2DM for the same duration. This might be one of the possible reasons why there is only a very limited number of published data about the relationship between worse CRC survival and the duration of T2DM which may also be one of the reasons for the difficulty of verification. Previously, it has been reported that a T2DM duration of over 10 years has significantly increased the risk for shorter DSS and OS, but a less than 10 years duration of T2DM was only associated with an increased risk for worse DSS but not for OS^10^. Similar results have been obtained by Yuan et al.^11^, although the median survival was very similar in both sexes for the > 10 years T2DM duration cohort, they could justify the significant effect of T2DM duration > 10 years only in women. It has to be noted, that, unfortunately, they have not performed the direct comparison of the two T2DM cohorts and the diabetes groups were compared to the non-T2DM participants only^11^. The univariate effect of T2DM has not been justified for OS nor for DSS in the study of Amshoff et al.^10^, while in the study of Yuan et al.^11^ mixed results have been found for the different sub-cohorts: the most significant survival worsening effect has been observed for those women with longer survival times (> 5 years) ^11^. Two further studies investigated whether pre-existing or incidental/later T2DM might affect the survival of CRC patients^8,9^. While Qiang et al.^9^ have found a significantly higher risk for those patients with a pre-existing disease, no such result has been reported in the study of Teo et al.^8^. In addition, Tao et al.^8^ have also investigated the effect of various T2DM durations (0–5 years, 5–10 years and > 10 years) over CRC survival, but in contrast to the Amshoff^10^ and Yuan^11^ studies they have reported no effect of T2DM duration over DSS, while a marginal and significant increase in OS have been observed in the “5–10 years” and “> 10 years” cohorts, respectively^8^. In the current study, the results were independent of age and various comorbidities (except for hypertension), and more similar to the first two aforementioned studies^10,11^: T2DM alone has less effect on the survival of patients, however, when the study participants were grouped into T2DM-duration categories, the survival time of T2DM patients with shorter T2DM durations was significantly longer. It has to be highlighted, that both in univariate and in multivariate settings, in most cases both OS and DSS was significantly affected by the shorter duration of T2DM, or at least the same tendencies (e.g., similar HRs but marginal 95% CIs) were found in the two survival indicators. This observation of our study is somewhat in line, but mostly in contrast to some recent literature^14,15^. Both Backer et al.^15^ and Li et al.^14^ reported that CRC patients with T2DM have an increased risk for all-cause mortality, but not for DSS/cancer-specific mortality. It has to be noted, however, that in both studies the HRs for DSS were similar to that of OS, however, 95% CIs included HR = 1, but their range was in the higher risk range for both articles. One hypothesis^14,15^ regarding this controversy is that T2DM is a complex disease that is significantly affected by various comorbidities^4^, which can also influence the effectiveness of oncological treatments, or even their applicability^15,22–25^. The tendency towards the increased risk for shorter DSS in the two meta-analyses^14,15^, and the results of the current study suggests that there might be more behind this than just the increased risk caused by more frequent comorbidities. Due to the limiting factors of the current study, such as the retrospective design, this question cannot be answered in more depth and further studies are required.

Further novel additions to the literature of this study were, that we were able to justify the significant effect of T2DM durations over CRC survival in specialized, longitudinal survival models as well. We could also confirm that in most comparisons the ‘longer than’ cohorts had worse survival than the ‘shorter than’ cohorts. Moreover, the survival curves of the ‘shorter than’ cohorts were very similar to that of those CRC patients without T2DM. It has to be noted, that none of these observations could be justified for the ‘≤ 20 years’ and ‘> 20 years’ cohorts, which is with high probability due to the low number of patients who had a T2DM duration > 20 years. Furthermore, using conditional survival methods we could demonstrate how the survival of the patients with different T2DM durations changes. In the first 3 years after CRC diagnosis the best OS could be found in those patients with a T2DM duration of fewer than 5 years, but after the 3rd year after CRC diagnosis this advantage disappeared, and all T2DM sub-cohorts had basically the same survival curve in the later times. It should be noted, however, that if staging was added in multivariate settings, its effect cancelled out every other explanatory variable, including the T2DM durations.

HbA_1C_ is one of the best markers for patient adherence^27^. Most guidelines^12,28^ agree on that a target HbA_1C_ of 7.0% is the most optimal to reach the best glycemic control. It has to be noted, however, that too low HbA_1C_ is also disadvantageous, as frequent hypoglycemia can also increase the cardiovascular risk of patients^29^, therefore a personalized treatment target range of 6.0–8.0% was also suggested previously^12^. Based on our result that HbA_1C_ of the study subjects was constant throughout the study and within the treatment target range of the Hungarian guidelines^12^, we hypothesize that patient adherence was adequate enough throughout the study in the T2DM cohort, and the significant effect of T2DM duration over CRC survival is with high probability unaffected by adherence to treatment.

Patients of the non-T2DM CRC group were significantly younger than those within the T2DM group (64.4 vs. 67.9 years). Over the age of 60 years, the incidence of T2DM is significantly increased, almost every fifth person is estimated to have diabetes^2^. In Hungary, based on data from prescribed antidiabetic medicines, ~ 20% of the population over 60 years has T2DM, however, no data are available on the number of T2DM patients who do not redeem their prescription or who are only on diet. Therefore, the actual number of T2DM patients in this age group is expected to be much higher, every fourth or third elderly person in Hungary may have diabetes^30^. Moreover, further analysis of our data revealed that patients under the age of 50 years were more common in the non-T2DM group. It is known that both the incidence of CRC^31,32^ and T2DM^33^ rises within the young adults. Although these trends are also true for Hungary, we can only speculate why the number of patients younger than 50 years of age did not appear in higher numbers among the CRC + T2DM patients. Further investigation of this question is required.

Further goals of the current research were to compare how various laboratory parameters change with the course of CRC in the different cohorts. It was found that, as somewhat expected, fasting plasma glucose was constantly higher in the T2DM cohort. Total, HDL, and LDL cholesterol levels were constantly lower in the T2DM cohort. The latter observation is due to the higher incidence of cardiovascular co-morbidities and in line with that, the higher usage of lipid-lowering agents in T2DM^34^. The observed lower HDL level of T2DM patients has to be highlighted; while lower total and LDL cholesterol levels are considered beneficial, lower HDL cholesterol is usually associated with an increased risk of various cardiovascular diseases^35^. It was also observed that the eGFR of T2DM patients decreased with the course of the disease, while no such trend could be observed in those CRC patients without T2DM. The constant decrease of eGFR with the progression of T2DM is well known^4,36^. The duration of diabetes significantly correlates with the decrease in eGFR and the increase in chronic kidney disease^37^. The latter could be also observed in our data. The lower hemoglobin and hematocrit levels observed in those with longer T2DM duration are—with high probability—also associated with this observation as anemia(-like) symptoms are indirect signs of eGFR decline^38^. Despite the similarity of the T2DM and non-T2DM cohorts (as shown in Table 1), all the laboratory deviations and survival differences presented here highlight that those CRC patients, who are also suffering from T2DM are more vulnerable. It is hypothesized that these patients require more attention from the practicing oncologist and with a high probability, a close cooperation between oncologists and diabetologists may be necessary.

Based on all of the results presented above, the following hypothesis can be assumed. T2DM is known as a progressive disease^4,7^, which is associated with various micro- and macrovascular complications, including but not limited to angiopathy, the more frequent occurrence of hypertension and mCV events (myocardial infarction, stroke, etc.), renal failure, etc. Both CRC^39,40^ and T2DM^40,41^ can cause dysfunction of the immune system and impair cytokine production resulting in an immunosuppressed state. Including the latter, it has been suggested that several diabetes-related changes can promote cancer and the faster development of advanced stages^16–20^. Although most changes occurring in the tumor microenvironment can be counteracted with appropriate treatments^42^, the proper oncological treatment of CRC patients with T2DM might be also impaired^22–25^ due to the disease worsening effects of T2DM, which can ultimately further increase the risk for shorter survival times of this patient population. Our finding that CRC patients with T2DM, who have had T2DM for a longer period of time have worse survival is in line with the above detailed literature data. Our novel result that CRC patients with a T2DM duration of < 5 years have better survival in the first 3 years post-CRC diagnosis, then all CRC + T2DM patients had the same survival independent of their T2DM duration fits into our existing knowledge. Unfortunately, we could not investigate in the current study, what might be behind this observation, and we can only speculate whether it is influenced by the various T2DM comorbidities, molecular changes caused by T2DM, the increased number of side effects of cancer therapy in this population, etc. Although the exact mechanisms are not yet known, due to the vulnerability of this patient population we suggest that those CRC patients with T2DM need closer monitoring, both by the oncologist and the diabetologist. It is necessary to achieve the best possible, personalized adherence to T2DM therapy in order to minimize the development of diabetic complications, which has to be adjusted for every patients’ age, general condition and T2DM education level/understanding.

## Limitations of the study

The study had some limitations, including the retrospective study design, the heterogeneity of patients, and that some parameters were not available for all the patients (e.g., the duration of T2DM was missing for 44 study subjects). A further limitation of the study was that in the case of joint and conditional survival modeling DSS can be computed currently only in a somewhat biased way. This yields a slightly overestimated result; therefore, these results should be treated with some caution, and possibly a re-study of these questions may be necessary when the updated methods will become available. The number of patients with a T2DM duration > 20 years was low. Some laboratory parameters were not available for every visit, and due to the lower number of long survivors, measurements at later time points were less frequent. Therefore, to reduce the resulting biases, we chose statistical methods that can robustly address the problem of missing values^43^. Furthermore, no multivariable analysis could be performed due to their technical limitations. It must be also mentioned that at the time of CRC diagnosis of the patients, KRAS, NRAS, and BRAF pathway analysis, and further molecular profiling of the tumors were performed only when it was necessary, and due to this, no analysis on tumor-related genetic alterations could be tested.

## Conclusion

In summary, a retrospective observational study was conducted to evaluate the effect of T2DM duration on CRC survival. Evidence suggests that both DSS and OS are influenced by different durations of T2DM, which is most likely free from the confounding effect of treatment adherence (with a high probability). Furthermore, it was found that within the first three years after CRC diagnosis, those patients with longer T2DM durations have an increased risk for shorter survival times, with which more pathological laboratory findings were found simultaneously. In general, more pathological laboratory results were confirmed in the T2DM cohorts, which indicates an increased vulnerability of these patients. Based on the results of the current study, increased monitoring of this patient group is recommended, especially within the first 3 years post-CRC diagnosis.

## Methods

The study was approved by the Regional and Institutional Committee of Science and Research Ethics, Semmelweis University (SE TUKEB 21-14/1994, approval date of latest modification: February 23, 2021), and conducted in concordance with the WMA Declaration of Helsinki. Patient consent was approved to be waived due to the retrospective, anonymous data collection nature of the study. Handling of patient data was in accordance with the General Data Protection Regulation issued by the European Union.

### Patients and study design

A retrospective longitudinal observational study was conducted with the inclusion of 817 CRC patients, who attended the Department of Internal Medicine and Hematology, Semmelweis University, Budapest, and at the Department of Internal and Medicine and Oncology, Semmelweis University, Budapest, between 2006 and 2018. All patient data was obtained anonymously from the medical database of Semmelweis University. The exclusion criteria included age < 18 years, any previous malignancies, known inflammatory bowel disease, mental disease, hematologic disease, and/or systemic autoimmune disease, and patients with an Eastern Cooperative Oncology Group (ECOG) performance status > 2.

Of the 817 patients, a total of 4543 laboratory measurements were recorded as follows. A baseline visit was performed at the time of tumor diagnosis, prior to any surgical and/or oncological interventions. Later visits were done 4–6 weeks after primary tumor resection (if feasible), and every 6 months thereafter.

### Clinicopathological and laboratory data measurements

Disease history data including co-morbidities and recent medications were collected. Laboratory results of fasting blood samples were recorded for every visit. Complete blood count, liver enzymes, creatinine level, plasma glucose, lipids, high-sensitivity C-reactive protein, HbA_1C,_ and CRC-related tumor markers were determined at the Central Laboratory of Semmelweis University, Budapest, Hungary. The eGFR was calculated using the CKD-EPI equations^44^.

T2DM was defined as follows. The presence of pre-existing diabetes was obtained from either medical history or from the current medication list of patients [e.g., metformin, acarbose, insulin(s), etc.] if the disease was not indicated in the medical history specifically. Incidental diabetes was defined as described by national guidelines^12^. (1) In the presence of classic diabetes symptoms^12,13^: fasting and random postprandial glucose values are ≥ 7.0 mmol/L and ≥ 11.1 mmol/L; (2) in the absence of classic diabetes symptoms^12,13^: fasting and 2-h glucose values are ≥ 7.0 mmol/L and ≥ 11.1 mmol/L during an oral glucose tolerance test; or (3) HbA_1C_ ≥ 6.5%^12^. The duration of T2DM was calculated from the year of T2DM diagnosis in years. In those cases where T2DM was diagnosed at the same time or after the diagnosis of the tumor, zero or negative values (in years) were used at the time of tumor diagnosis, respectively. Moreover, the duration of T2DM was also recorded as the following dummy variables: T2DM duration is (1) > 5 years or ≤ 5 years; (2) > 10 years or ≤ 10 years; (3) > 15 years or ≤ 15 years; and (4) > 20 years or ≤ 20 years.

The tumor staging was given by histopathological examination of surgical specimens (if feasible) and imaging studies. The 8th Edition of the American Joint Committee on Cancer Staging was used^45^. The sidedness of the tumor was described as detailed by Baran et al.^46^ Due to the large number of combinations in patients’ chemotherapeutic treatments, the lineage number of the final treatment the patient received was recorded. In short, patients received a cytotoxic doublet with a biological agent (bevacizumab or anti-EGFR recombinant chimeric monoclonal antibody) as first-line and second-line treatment, and irinotecan + cetuximab and regorafenib or trifluridine/tipiracil were administered as third-line or above, as per ESMO and national guidelines^47–49^. DSS and OS were calculated as the time elapsed between the date of tumor diagnosis and cancer-related or any death, respectively. Patients alive at the time of study termination were right-censored, and the follow-ups of patients were terminated on February 28, 2023.

### Statistical analysis

Statistical analyses were performed using the R for Windows 4.2.3 environment (R Foundation for Statistical Computing, 2023, Vienna, Austria). Group comparisons were performed with Welch’s test, Fisher’s exact test, and the Cochran–Mantel–Haenszel chi-squared test. Longitudinal modeling of laboratory results was performed using natural cubic spline adjusted linear mixed-effects models (R library nlme, version 3.1-162). A value of *P* < 0.05 was considered statistically significant and the Holm method^50^ was used for the multiple comparisons problems. Continuous and count data were expressed as mean ± standard deviation and as the number of observations (percentage), respectively.

Patient survival was analyzed using three approaches, of which the second and third approach was only performed with the data of CRC patients having T2DM. First, OS and DSS were analyzed using Cox regression and cause-specific competing risk models (R library survival, version 3.5-5), respectively. Second, the longitudinal change in the occurrence of 5/10/15/20-year-long T2DM duration was analyzed using Bayesian univariate joint models (R library rstanarm, version 2.21.3), where the association structure was based on the current value of the linear predictor in the longitudinal sub-model. And third, using conditional survival models (R library condSURV, version 2.0.4) it was further investigated what additional information can be gained about the relationship between CRC survival and the longitudinal changes in the occurrence of 5/10/15/20-year-long T2DM duration. “Conventional” and joint model survival data was expressed as a hazard ratio (HR) and its 95% confidence/credible interval, while conditional survival data were expressed as the x-year survival rate and its 95% confidence interval. Naïve Kaplan–Meier-type plots and forest plots were drawn with the survminer (version 0.4.9) and forestplot (version 3.1.1) R packages, respectively.

### Supplementary Information

Supplementary Information.

## Data Availability

The datasets generated during and/or analyzed during the current study are available from the corresponding author on reasonable request.

## References

[1] Sung H (2021). Global Cancer Statistics 2020: GLOBOCAN estimates of incidence and mortality worldwide for 36 cancers in 185 countries. CA Cancer J. Clin..

[2] International Diabetes Federation. *IDF Diabetes Atlas* 10th edn (International Diabetes Federation, 2021).

[3] Shlomai G, Neel B, LeRoith D, Gallagher EJ (2016). Type 2 diabetes mellitus and cancer: The role of pharmacotherapy. J. Clin. Oncol..

[4] DeFronzo RA (2015). Type 2 diabetes mellitus. Nat. Rev. Dis. Primers.

[5] Gonzalez N (2017). 2017 Update on the relationship between diabetes and colorectal cancer: Epidemiology, potential molecular mechanisms and therapeutic implications. Oncotarget.

[6] Petrelli F (2021). Survival of colorectal cancer patients with diabetes mellitus: A meta-analysis. Can. J. Diabetes.

[7] Fonseca VA (2009). Defining and characterizing the progression of type 2 diabetes. Diabetes Care.

[8] Tao H (2020). Pre- and post-diagnosis diabetes as a risk factor for all-cause and cancer-specific mortality in breast, prostate, and colorectal cancer survivors: A prospective cohort study. Front. Endocrinol..

[9] Qiang JK (2020). Impact of diabetes on colorectal cancer stage and mortality risk: A population-based cohort study. Diabetologia.

[10] Amshoff Y (2018). Type 2 diabetes and colorectal cancer survival: The multiethnic cohort. Int. J. Cancer.

[11] Yuan C (2021). Preexisting type 2 diabetes and survival among patients with colorectal cancer. Cancer Epidemiol. Biomarkers Prev..

[12] Gaál Z (2020). Clinical Practice Guideline—Diagnosis of diabetes, and antihyperglycaemic treatment and care of patients with diabetes in adulthood. Diabetol Hung..

[13] ElSayed NA (2023). 2. Classification and diagnosis of diabetes: Standards of care in diabetes-2023. Diabetes Care.

[14] Li J, Liu J, Gao C, Liu F, Zhao H (2017). Increased mortality for colorectal cancer patients with preexisting diabetes mellitus: An updated meta-analysis. Oncotarget.

[15] Becker DJ (2020). Diabetes mellitus and colorectal carcinoma outcomes: A meta-analysis. Int. J. Colorectal. Dis..

[16] Le Marchand L, Wilkens LR, Kolonel LN, Hankin JH, Lyu LC (1997). Associations of sedentary lifestyle, obesity, smoking, alcohol use, and diabetes with the risk of colorectal cancer. Cancer Res..

[17] Nunez C, Nair-Shalliker V, Egger S, Sitas F, Bauman A (2018). Physical activity, obesity and sedentary behaviour and the risks of colon and rectal cancers in the 45 and up study. BMC Public Health.

[18] de Kort S (2019). Diabetes mellitus, genetic variants in the insulin-like growth factor pathway and colorectal cancer risk. Int. J. Cancer.

[19] Giovannucci E (2010). Diabetes and cancer: A consensus report. Diabetes Care.

[20] Lundby A (2015). Surface-expressed insulin receptors as well as IGF-I receptors both contribute to the mitogenic effects of human insulin and its analogues. J. Appl. Toxicol..

[21] Overbeek JA (2019). Sex- and site-specific differences in colorectal cancer risk among people with type 2 diabetes. Int. J. Colorectal. Dis..

[22] Gross CP, McAvay GJ, Guo Z, Tinetti ME (2007). The impact of chronic illnesses on the use and effectiveness of adjuvant chemotherapy for colon cancer. Cancer.

[23] Zanders MM (2013). Diminishing differences in treatment between patients with colorectal cancer with and without diabetes: A population-based study. Diabet. Med..

[24] Maas HAAM (2011). The effects of age and comorbidity on treatment patterns for radiotherapy and survival in patients with mobile rectal cancer: A population-based study. Eur. Geriatr. Med..

[25] Lemmens VE (2005). Co-morbidity leads to altered treatment and worse survival of elderly patients with colorectal cancer. Br. J. Surg..

[26] Garcia-Perez LE, Alvarez M, Dilla T, Gil-Guillen V, Orozco-Beltran D (2013). Adherence to therapies in patients with type 2 diabetes. Diabetes Ther.

[27] Sherwani SI, Khan HA, Ekhzaimy A, Masood A, Sakharkar MK (2016). Significance of HbA1c test in diagnosis and prognosis of diabetic patients. Biomark Insights.

[28] ElSayed NA (2023). 6. Glycemic targets: Standards of care in diabetes-2023. Diabetes Care.

[29] Desouza CV, Bolli GB, Fonseca V (2010). Hypoglycemia, diabetes, and cardiovascular events. Diabetes Care.

[30] Kempler P (2016). Prevalence and financial burden of type 2 diabetes mellitus in Hungary between 2001–2014—Results of the analysis of the National Health Insurance Fund database. Diab Hung.

[31] Sinicrope FA (2022). Increasing incidence of early-onset colorectal cancer. N. Engl. J. Med..

[32] Siegel RL, Jakubowski CD, Fedewa SA, Davis A, Azad NS (2020). Colorectal cancer in the young: Epidemiology, prevention, management. Am. Soc. Clin. Oncol. Educ. Book.

[33] Xie J (2022). Global burden of type 2 diabetes in adolescents and young adults, 1990–2019: Systematic analysis of the Global Burden of Disease Study 2019. BMJ.

[34] ElSayed NA (2023). 10. Cardiovascular disease and risk management: Standards of care in diabetes-2023. Diabet. Care.

[35] Farbstein D, Levy AP (2012). HDL dysfunction in diabetes: Causes and possible treatments. Expert Rev. Cardiovasc. Ther..

[36] Bramlage P (2020). Renal function deterioration in adult patients with type-2 diabetes. BMC Nephrol..

[37] Joshi R (2023). Prevalence and risk factors of chronic kidney disease among patients with type 2 diabetes mellitus at a tertiary care hospital in Nepal: A cross-sectional study. BMJ Open.

[38] Xie L (2023). Anemia is a risk factor for rapid eGFR decline in type 2 diabetes. Front. Endocrinol..

[39] Greten FR, Grivennikov SI (2019). Inflammation and cancer: Triggers, mechanisms, and consequences. Immunity.

[40] Herczeg G (2022). Does diabetes affect paraneoplastic thrombocytosis in colorectal cancer?. Open Med.

[41] Berbudi A, Rahmadika N, Tjahjadi AI, Ruslami R (2020). Type 2 diabetes and its impact on the immune system. Curr. Diabet. Rev..

[42] Wozniakova M, Skarda J, Raska M (2022). The role of tumor microenvironment and immune response in colorectal cancer development and prognosis. Pathol. Oncol. Res..

[43] Ibrahim JG, Molenberghs G (2009). Missing data methods in longitudinal studies: A review. Test (Madr).

[44] Schwandt A (2017). Comparison of MDRD, CKD-EPI, and Cockcroft–Gault equation in relation to measured glomerular filtration rate among a large cohort with diabetes. J. Diabet. Complicat..

[45] Jessup J, Amin M (2018). Colon and rectum. AJCC Cancer Staging Manual.

[46] Baran B (2018). Difference between left-sided and right-sided colorectal cancer: A focused review of literature. Gastroenterol. Res..

[47] Argiles G (2020). Localised colon cancer: ESMO Clinical Practice Guidelines for diagnosis, treatment and follow-up. Ann. Oncol..

[48] Glynne-Jones R (2017). Rectal cancer: ESMO Clinical Practice Guidelines for diagnosis, treatment and follow-up. Ann Oncol.

[49] Van Cutsem E (2016). ESMO consensus guidelines for the management of patients with metastatic colorectal cancer. Ann. Oncol..

[50] Holm S (1979). A Simple sequentially rejective multiple test procedure. Scand. J. Stat..

