# Supplementary material for: Influence of the duration of type 2 diabetes mellitus on colorectal cancer outcomes
Source: Sci Rep. 2023 Aug 10;13:12985. doi: 10.1038/s41598-023-40216-3 (PMC10415401; doi:10.1038/s41598-023-40216-3)
Supplement: Supplementary file 1 — Supplementary Information. [file 41598_2023_40216_MOESM1_ESM.pdf]

Influence of the duration of type 2 diabetes mellitus on colorectal cancer outcomes

Magdolna Herold, Attila Marcell Szasz, Gyongyver Szentmartoni, Emoke Martinek, Viktor Madar-Dank, Andras Jozsef Barna, Reka Mohacsi, Aniko Somogyi, Magdolna Dank and Zoltan Herold

Table S1. Results of survival analyses comparing the type 2 diabetes mellitus (T2DM) sub-cohorts with different T2DM durations.

Groups compared	Model	Hazard rate	95% CI	*p*-Value
T2DM ≤ 5 years (ref.) vs. T2DM > 5 years	DSS	1.718	1.053 – 2.804	0.030
	OS	1.794	1.159 – 2.778	0.009
T2DM ≤ 10 years (ref.) vs. T2DM > 10 years	DSS	1.703	1.056 – 2.744	0.029
	OS	1.540	0.984 – 2.410	0.059
T2DM ≤ 15 years (ref.) vs. T2DM > 15 years	DSS	1.992	1.179 – 3.365	0.010
	OS	1.755	1.033 – 2.982	0.038
T2DM ≤ 20 years (ref.) vs. T2DM > 20 years	DSS	1.334	0.514 – 3.463	0.554
	OS	1.003	0.439 – 2.308	0.994

CI: confidence interval; DSS: disease-specific survival; OS: overall survival; ref: reference category.

Table S2. Results of survival analyses comparing the colorectal patients (CRC) without type 2 diabetes mellitus (T2DM), and the CRC + T2DM sub-cohorts with different T2DM durations.

Groups compared	Model	Hazard rate	95% CI	*p*-Value
CRC only (ref.) vs. CRC + T2DM ≤ 5 years	DSS	0.742	0.513 – 1.071	0.111
	OS	0.802	0.576 – 1.116	0.190
CRC only (ref.) vs. CRC + T2DM > 5 years	DSS	1.175	0.839– 1.644	0.349
	OS	1.377	1.043 – 1.816	0.024
T2DM ≤ 5 years (ref.) vs. T2DM > 5 years	DSS	1.584	0.984 – 2.550	0.058
	OS	1.248	1.142 – 2.582	0.009
CRC only (ref.) vs. CRC + T2DM ≤ 10 years	DSS	0.811	0.593 – 1.110	0.191
	OS	0.939	0.717 – 1.229	0.647
CRC only (ref.) vs. CRC + T2DM > 10 years	DSS	1.299	0.869 – 1.941	0.202
	OS	1.420	1.004 – 2.008	0.048
T2DM ≤ 10 years (ref.) vs. T2DM > 10 years	DSS	1.601	0.984 – 2.603	0.058
	OS	1.512	0.998 – 2.291	0.051
CRC only (ref.) vs. CRC + T2DM ≤ 15 years	DSS	0.826	0.614 – 1.110	0.204
	OS	0.961	0.748 – 1.235	0.757
CRC only (ref.) vs. CRC + T2DM > 15 years	DSS	1.572	1.010 – 2.447	0.045
	OS	1.654	1.085 – 2.522	0.019
T2DM ≤ 15 years (ref.) vs. T2DM > 15 years	DSS	1.905	1.143– 3.174	0.013
	OS	1.721	1.075 – 2.756	0.024
CRC only (ref.) vs. CRC + T2DM ≤ 20 years	DSS	0.928	0.710 – 1.214	0.587
	OS	1.067	0.847 – 1.343	0.583
CRC only (ref.) vs. CRC + T2DM > 20 years	DSS	1.185	0.538 – 2.611	0.673
	OS	1.132	0.531 – 2.411	0.749
T2DM ≤ 20 years (ref.) vs. T2DM > 20 years	DSS	1.277	0.563 – 2.895	0.559
	OS	1.061	0.487 – 2.310	0.882

CI: confidence interval; DSS: disease-specific survival; OS: overall survival; ref: reference category.

Table S3. *P*-values of multivariate survival analyses comparing the type 2 diabetes mellitus (T2DM) sub-cohorts with different T2DM durations.

Groups compared	Survival type	Model I	Model II	Model III	Model IV
T2DM ≤ 5 years (ref.) vs. T2DM > 5 years	DSS	0.011	–	–	–
	OS	0.033	–	–	–
T2DM ≤ 10 years (ref.) vs. T2DM > 10 years	DSS	–	0.023	–	–
	OS	–	0.174	–	–
T2DM ≤ 15 years (ref.) vs. T2DM > 15 years	DSS	–	–	0.017	–
	OS	–	–	0.146	–
T2DM ≤ 20 years (ref.) vs. T2DM > 20 years	DSS	–	–	–	0.586
	OS	–	–	–	0.863
Age (years)	DSS	0.197	0.292	0.419	0.711
	OS	0.601	0.333	0.246	0.152
Hypertension [No (ref.) vs. Yes]	DSS	0.302	0.238	0.304	0.239
	OS	0.363	0.326	0.382	0.324
Major cardiovascular event(s) [a] prior to CRC [No (ref.) vs. Yes]	DSS	0.901	0.917	0.819	0.970
	OS	0.389	0.470	0.506	0.412
Thyroid disease [No (ref.) vs. Yes]	DSS	0.141	0.228	0.175	0.186
	OS	0.627	0.861	0.761	0.702
Appendectomy [No (ref.) vs. Yes]	DSS	0.346	0.356	0.210	0.304
	OS	0.579	0.604	0.457	0.534
Cholecystectomy [No (ref.) vs. Yes]	DSS	0.128	0.094	0.171	0.174
	OS	0.182	0.152	0.246	0.199

[a] Myocardial infarction, stroke, transient ischemic attack, pulmonary embolism, coronary artery bypass grafting, and/or stent implantation. DSS: disease-specific survival; OS: overall survival; ref: reference category.

Table S4. *P*-values of multivariate survival analyses comparing the colorectal patients (CRC) without type 2 diabetes mellitus (T2DM), and the CRC + T2DM sub-cohorts with different T2DM durations.

Groups compared	Model I	Model II	Model III	Model IV
**Disease-specific survival**				
CRC only (ref.) vs. T2DM ≤ 5 years	0.048	–	–	–
CRC only (ref.) vs. T2DM > 5 years	0.619	–	–	–
T2DM ≤ 5 years (ref.) vs. T2DM > 5 years	0.058	–	–	–
CRC only (ref.) vs. T2DM ≤ 10 years	–	0.076	–	–
CRC only (ref.) vs. T2DM > 10 years	–	0.329	–	–
T2DM ≤ 10 years (ref.) vs. T2DM > 10 years	–	0.049	–	–
CRC only (ref.) vs. T2DM ≤ 15 years	–	–	0.089	–
CRC only (ref.) vs. T2DM > 15 years	–	–	0.123	–
T2DM ≤ 15 years (ref.) vs. T2DM > 15 years	–	–	0.019	–
CRC only (ref.) vs. T2DM ≤ 20 years	–	–	–	0.286
CRC only (ref.) vs. T2DM > 20 years	–	–	–	0.863
T2DM ≤ 20 years (ref.) vs. T2DM > 20 years	–	–	–	0.0598
Age (years)	0.609	0.630	0.637	0.726
Hypertension [No (ref.) vs. Yes]	0.010	0.009	0.010	0.010
Major cardiovascular event(s) [a] prior to CRC [No (ref.) vs. Yes]	0.903	0.837	0.816	0.993
Thyroid disease [No (ref.) vs. Yes]	0.654	0.788	0.716	0.702
Appendectomy [No (ref.) vs. Yes]	0.818	0.866	0.743	0.829
Cholecystectomy [No (ref.) vs. Yes]	0.221	0.191	0.275	0.241
**Overall survival**				
CRC only (ref.) vs. T2DM ≤ 5 years	0.085	–	–	–
CRC only (ref.) vs. T2DM > 5 years	0.247	–	–	–
T2DM ≤ 5 years (ref.) vs. T2DM > 5 years	0.027	–	–	–
CRC only (ref.) vs. T2DM ≤ 10 years	–	0.256	–	–
CRC only (ref.) vs. T2DM > 10 years	–	0.265	–	–
T2DM ≤ 10 years (ref.) vs. T2DM > 10 years	–	0.091	–	–
CRC only (ref.) vs. T2DM ≤ 15 years	–	–	0.302	–
CRC only (ref.) vs. T2DM > 15 years	–	–	0.158	–
T2DM ≤ 15 years (ref.) vs. T2DM > 15 years	–	–	0.066	–
CRC only (ref.) vs. T2DM ≤ 20 years	–	–	–	0.700
CRC only (ref.) vs. T2DM > 20 years	–	–	–	0.933
T2DM ≤ 20 years (ref.) vs. T2DM > 20 years	–	–	–	0.971
Age (years)	0.103	0.085	0.080	0.063
Hypertension [No (ref.) vs. Yes]	0.004	0.004	0.005	0.005
Major cardiovascular event(s) [a] prior to CRC [No (ref.) vs. Yes]	0.609	0.634	0.631	0.488
Thyroid disease [No (ref.) vs. Yes]	0.944	0.915	0.996	0.977
Appendectomy [No (ref.) vs. Yes]	0.999	0.948	0.935	0.991
Cholecystectomy [No (ref.) vs. Yes]	0.257	0.235	0.331	0.286

[a] Myocardial infarction, stroke, transient ischemic attack, pulmonary embolism, coronary artery bypass grafting, and/or stent implantation.

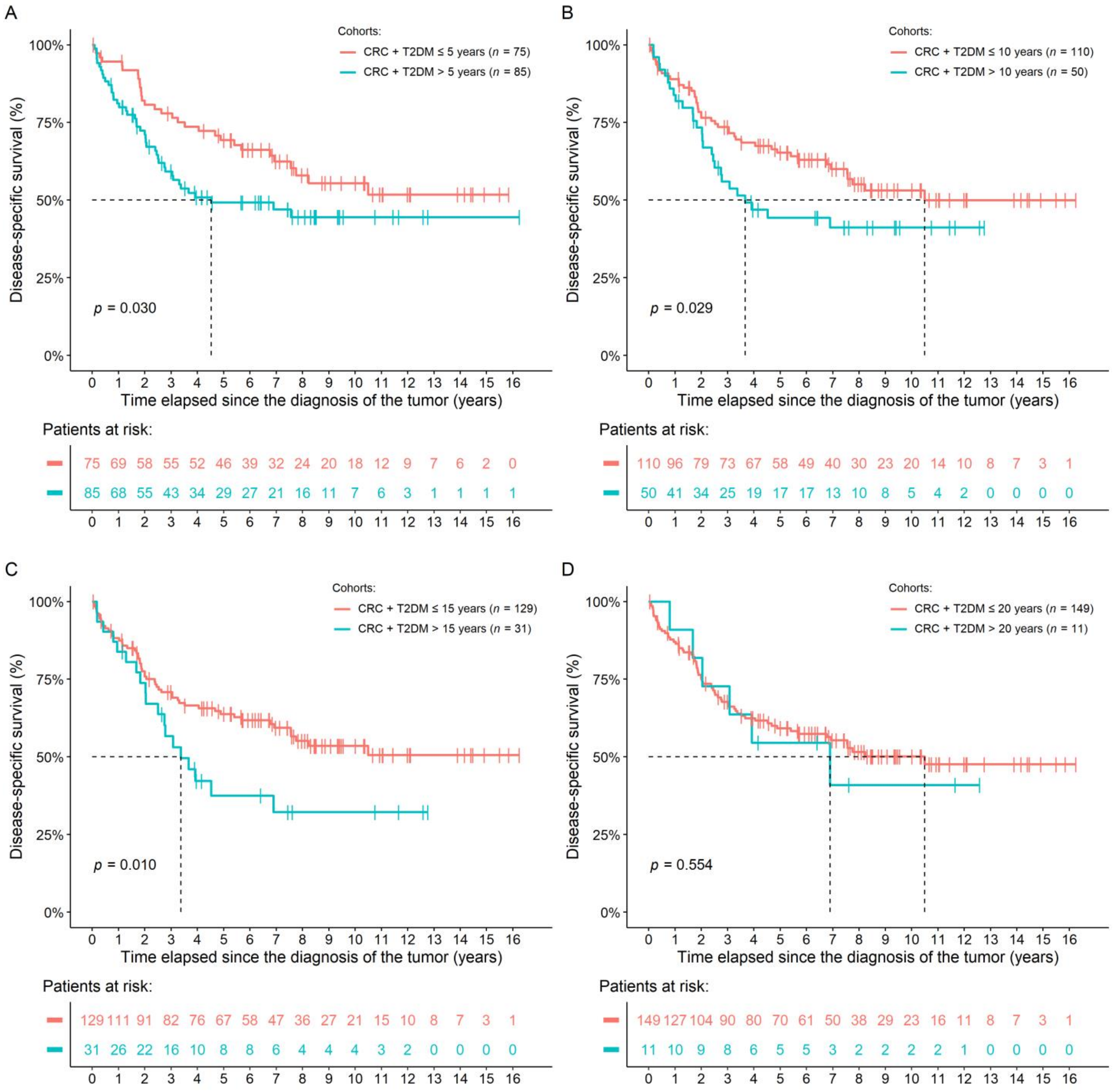

Figure S1. Differences in disease-specific survival of patients with a type 2 diabetes mellitus duration of (**A**) ≤ 5 years vs. > 5 years, (**B**) ≤ 10 years vs. > 10 years, (**C**) ≤ 15 years vs. > 15 years, and (**D**) ≤ 20 years vs. > 20 years. While in the first 3 comparisons patient survival was significantly (**A** and **C**) and marginally (**B**) worse, in the last comparison no difference could be justified.

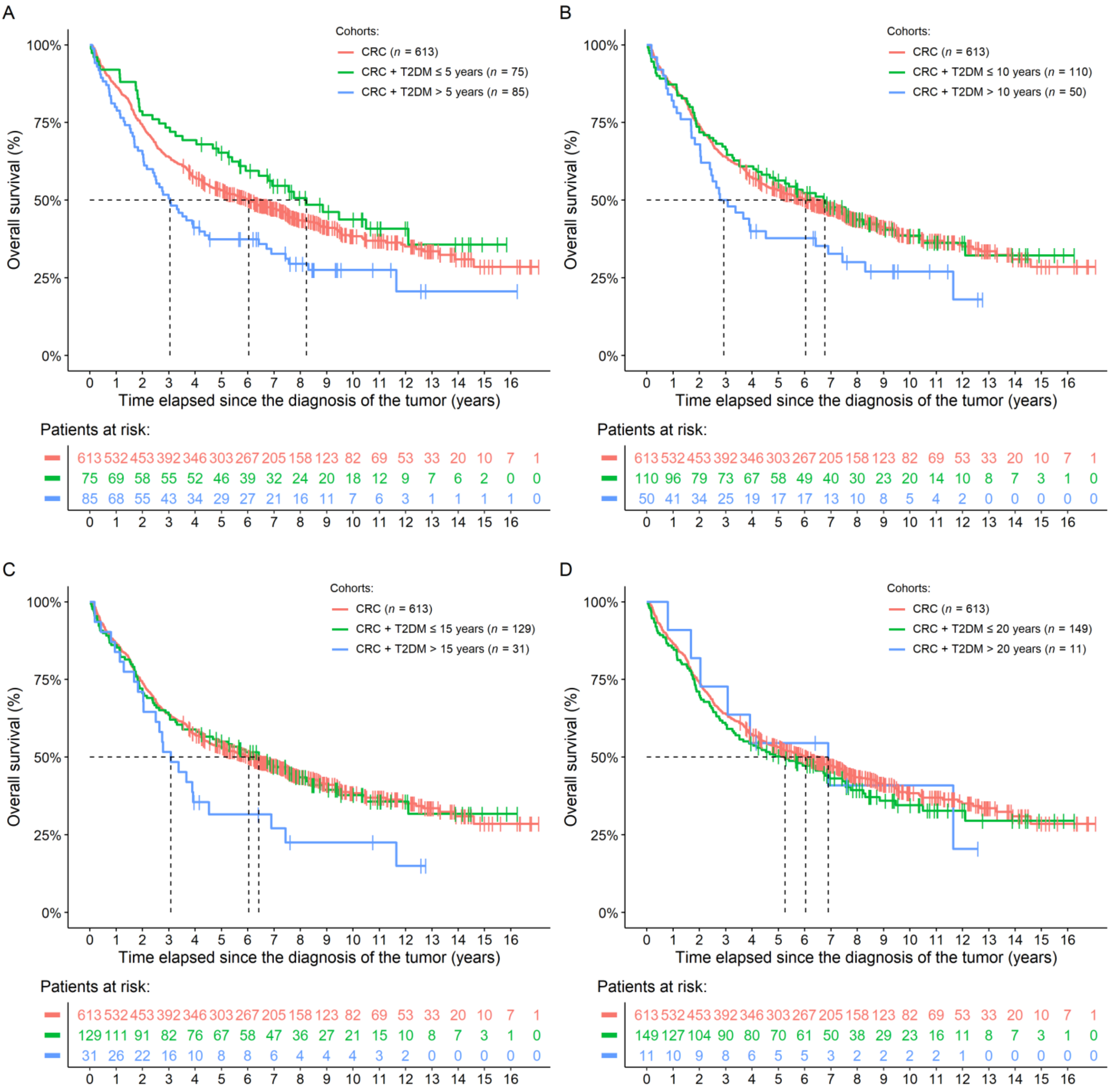

Figure S2. Differences in overall survival of patients with colorectal cancer (CRC) without type 2 diabetes mellitus (T2DM) and with a T2DM duration of (**A**) ≤ 5 years vs. > 5 years, (**B**) ≤ 10 years vs. > 10 years, (**C**) ≤ 15 years vs. > 15 years, and (**D**) ≤ 20 years vs. > 20 years. While in the first 3 comparisons patient survival was significantly worse in the "greater than" cohorts, in the last comparison no difference could be justified.

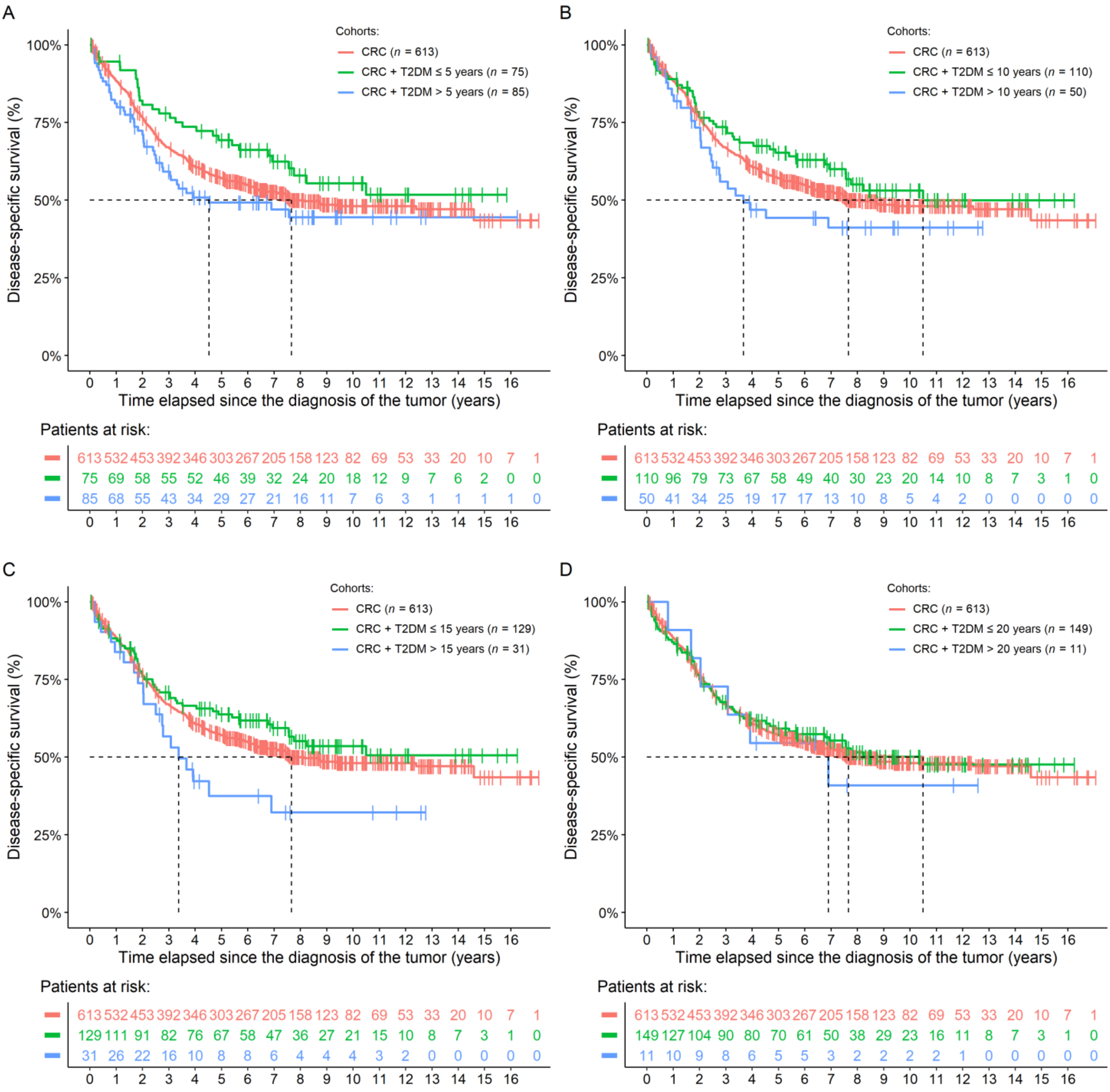

Figure S3. Differences in disease-specific survival of patients with colorectal cancer (CRC) without type 2 diabetes mellitus (T2DM) and with a T2DM duration of (**A**) ≤ 5 years vs. > 5 years, (**B**) ≤ 10 years vs. > 10 years, (**C**) ≤ 15 years vs. > 15 years, and (**D**) ≤ 20 years vs. > 20 years.

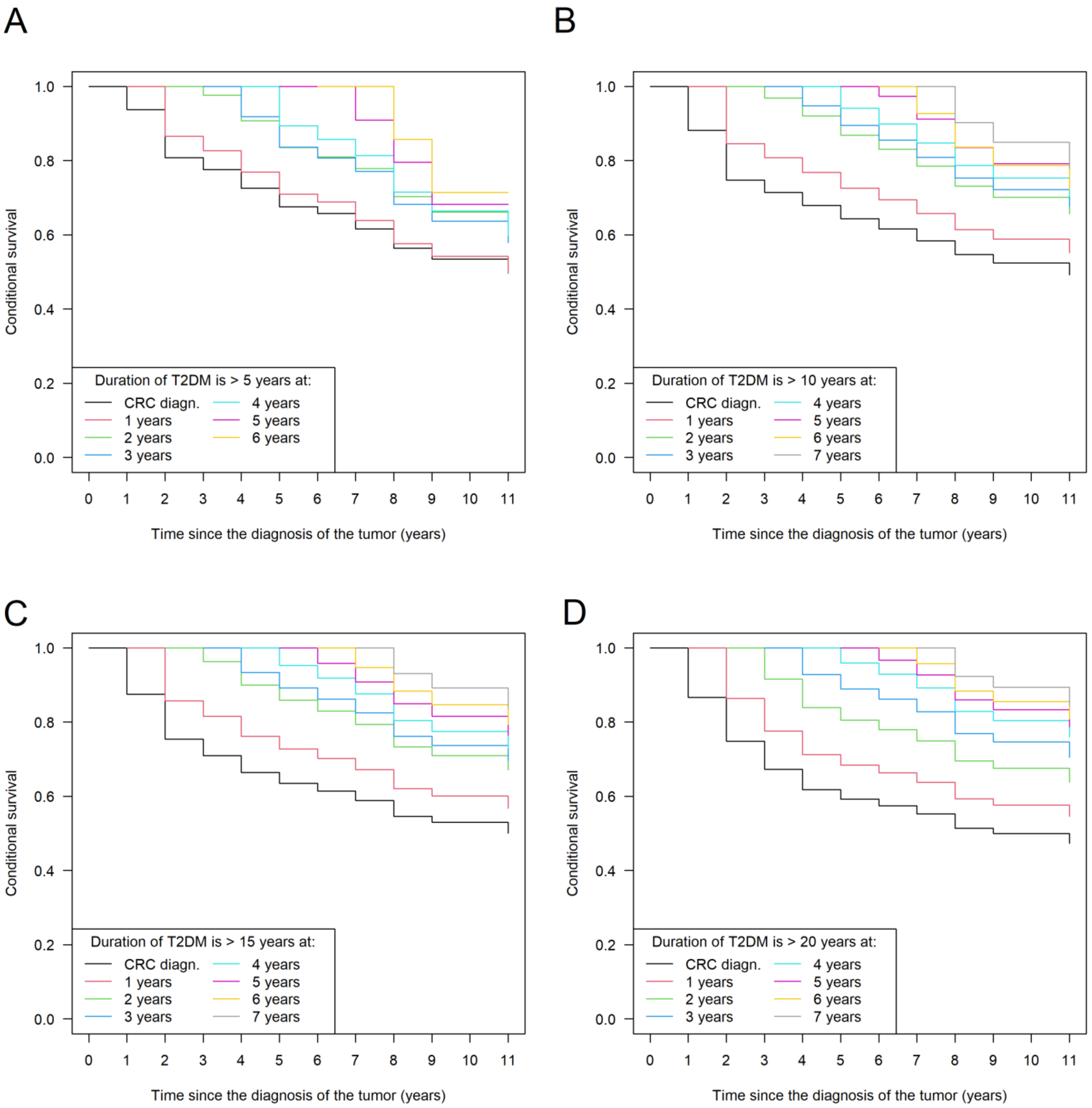

Figure S4. Conditional disease-specific survival of colorectal cancer (CRC) patients, who synchronously had type 2 diabetes mellitus (T2DM) for (**A**) > 5 years, (**B**) > 10 years, (**C**) > 15 years, and (**D**) > 20 years. Using this method, it was calculated whether the later the 5/10/15/20 years duration of T2DM occurs, the better the survival of the patients is.

Note: CRC patients without T2DM could not be included in this type of analysis.

Note 2: The results computed for disease-specific survival are somewhat distorted due to the lack of methods for competing risk calculation via joint survival modeling. Therefore, non-CRC related deaths were marked as censored events, which might have resulted in some overestimation of the HRs and their 95% CIs.

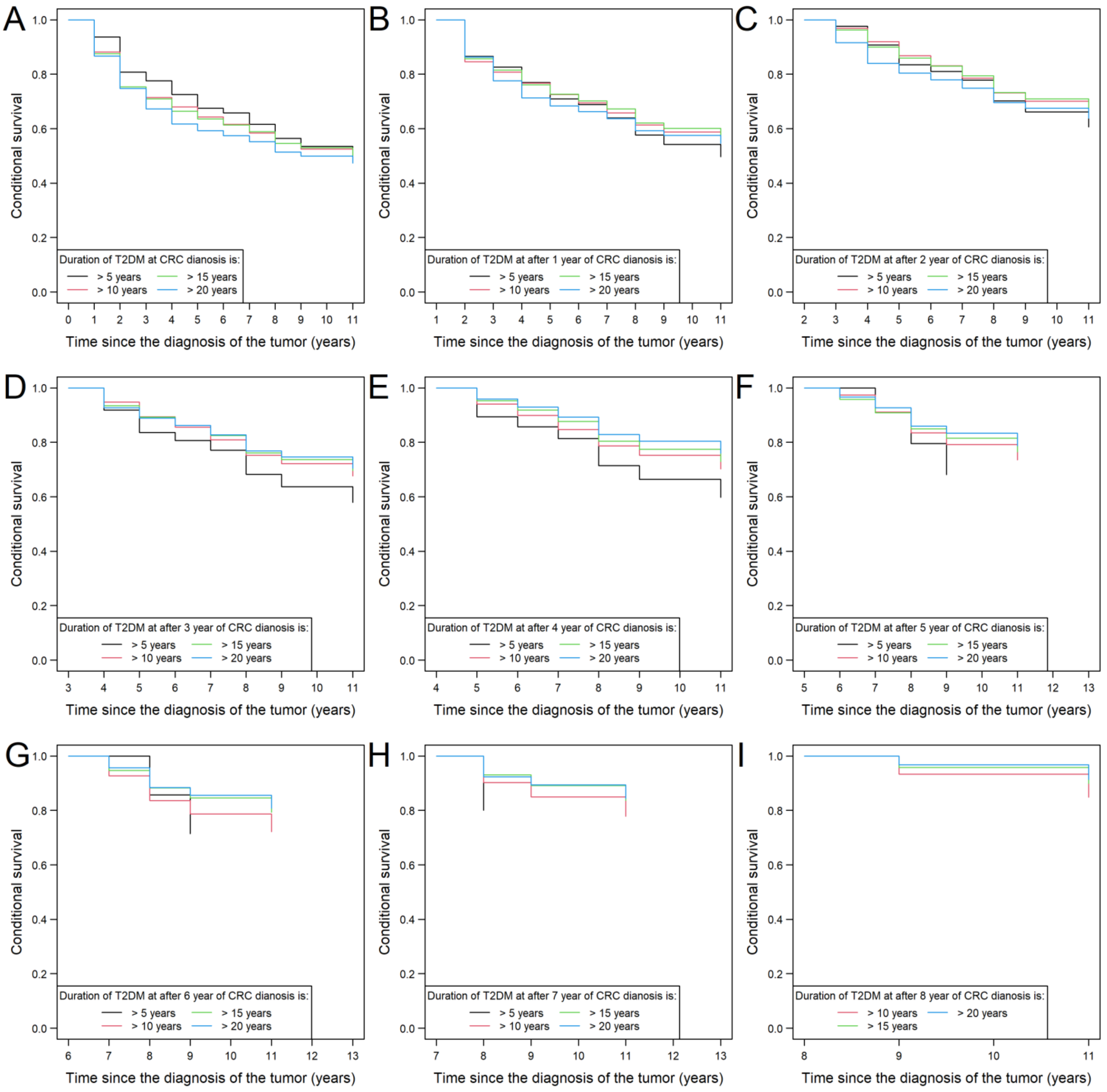

Figure S5. Conditional disease-specific survival of colorectal cancer (CRC) patients, who synchronously had type 2 diabetes mellitus (T2DM) for 5/10/15/20 years. By comparing the conditional survival curves of the 4 sub-cohorts it was found that around the 3rd year after the diagnosis of CRC, the positive effect of the shorter T2DM durations over patient survival became less prominent, and the four sub-cohorts are basically the same thereafter.

Note: CRC patients without T2DM could not be included in this type of analysis.

Note 2: The results computed for disease-specific survival are somewhat distorted due to the lack of methods for competing risk calculation via conditional survival modeling. Therefore, non-CRC related deaths were marked as censored events, which might have resulted in some overestimation of the HRs and their 95% CIs.
